# The Treatment of Acute Rheumatism

**Published:** 1893-04-08

**Authors:** 


### ST. MARY'S HOSPITAL.

### The Treatment of Acute Rheumatism.

The treatment of acute rheumatism in this article will be considered under the] following heads: (1) The general treatment of the disease. (2) The treatment of the complications.

*The General Treatment*.—The patient must be placed in a bed, with a firm but comfortable mattress, and should be well covered with bedclothes. The bed should be in such a position so as to be easily accessible from either side. A warm ward should be chosen, and the patient should be carefully protected from draughts; curtains may be placed round the head of the bed, if necessary. A flannel bedgown should be worn, and should be made so that the chest can be exposed without disturbing the patient. Should the pressure of the bedclothes give rise to pain or discomfort, they may be raised by means of a "cradle."

The affected joints should be carefully and gently sponged with warm water and soap, or with slightly alkalized warm water, and then wrapped up in several layers of cotton wool, secured by a few turns of an ordinary domette bandage. The cotton wool must be occasionally renewed. Should the measure fail to give relief to the pain, the joints may be wrapped in lint, previously soaked in an alkaline solution to which tinct. opii (ʒss to the ℥j) or ext. belladon. (gr. xv. to the ℥j) has been added. The lint may be covered with oiled silk, and the whole joint enclosed in cotton wool as above described. Thirst may be relieved by the free use of aërated alkaline waters, taken alone or mixed with milk or lemon-juice. Barley or toast-water may be given for the same purpose. The diet should consist of fluids, chiefly in the form of milk. Strong beef tea is better avoided, but a little weak chicken-broth may be given. The patient should be fed at short and regular intervals. As convalescence becomes established, custards or milk puddings, boiled fish, and lastly, fowl, may be added to the dietary. Meat must be entirely prohibited until the patient is perfectly free from all rheumatic symptoms.

Alcoholic stimulants, if required, should be given in the form of brandy. The amount prescribed must be regulated by the severity of the symptoms, but except in those cases attended by cardiac failure, it is rarely found necessary to exceed eight or twelve ounces in the twenty-four hours. Malt liquors and the stronger wines should be entirely avoided during convalescence.

It is the custom at St. Mary's to commence the medicinal treatment by the administration of a purge, a powder consisting of calomel (gr. ii) and pulv. jalap. co (ʒss) being the one generally used. This may be succeeded by a saline, should it be required. This is followed by a mixture containing salicylate of soda or salicin, in combination with an alkali, which may, at first, be given every two or three hours, the interval being regulated according to the effect produced. The following prescription is a favourite one at St. Mary's: ℞ Sodae salicyl., gr. xx.; pot. bicarb., gr. xx.; sodæ cit., gr. xx.; syr. limon, ʒj; aq. chlorof. ʒj.

The mixture is to be given every hour for six hours, and then three times a day. If necessary this procedure may be repeated after a lapse of twenty-four hours. If after a third trial no good effect is produced alkalies are to be tried.

The toxic effects, viz., cardiac depression, sickness, deafness, delirium, &c., which may follow the administration of the salicylates, can be partially, at least, obviated by the addition of the tincture of nux vomica (♏ v.—♏ viii.) to the above mixture.

Should this treatment be followed by cessation of pain and reduction of fever, it should be continued for some days after the temperature has become normal, otherwise there is great likelihood of a relapse. In those cases in which the administration of salicylates fail to produce beneficial results the alkalies must be tried. The following is a convenient and pleasant mode of giving them: ℞ (A) Acid citric, gr. xv.; quin. sulph., gr. iii.; aq. chlorof ad., ℥j.; ℞ (B) pot. bicarb., gr. xx.; pot. citrat., gr. xx.; Syr. limon., ʒss.; aq.chlorof. ad, ℥j. § One ounce of each to be taken mixed every four hours. Salicin may be added to the mixture (B) if desirable, and occasionally the addition of sodæ cit. (ʒss.) to mixture (A) is found useful. The same precaution with regard to the continuance of the administration of this remedy applies as in the case of the salicylate mixture. Sleeplessness, at any stage of the disease, may be treated with pulv. ipecac. co.

The following mixture may sometimes be found useful in those cases in which pain is a predominant feature of the attack: ℞ pot. bicarb., gr. xxx.; tr. opii m. xv.; inf. buchu., ℥j, and it may be given every four hours until the pain is relieved.

The patient should on no account be allowed to leave his bed until at least a week or ten days after the temperature has become normal, even in the absence of cardiac complications. Wool should be worn next the skin, and all exposure to cold and draughts carefully avoided. After the temperature has been normal for a few days it is advisable to give iron with an alkali in place of the salicylate mixture.

The milder forms of iron are to be preferred at first, such as the ferri. et quin. cit. (gr. x), with pot. citrat. (gr. xv.), or the ferri et pot. tart. (gr. x.), with pot. bicarb. (gr. xx.) Later tinct. ferri perchlor. may be prescribed, with a few minims (1-4) of liq. arsenicalis.

The bowels during convalescence should receive careful attention. Should the joints continue stiff or swollen, they may be treated locally by means of small blisters, or painted with iodine. Massage is also useful.

A mixture, containing iodide of potassium (gr. v.), with mist. guaiaci. (℥j.) may be given, with a view of helping absorption. Change of air to a warm climate is also of much service.

*The Treatment of the Complications.*—The most dangerous but fortunately a rare complication of acute rheumatism is hyperpyrexia. Its onset is indicated by diminution of joint pain, with cessation of sweating, and general excitement, going on to delirium. Should these symptoms occur the temperature must be taken every half-hour, and if it rises to 105 deg. the patient must be placed in a bath at 98 deg., which is to be cooled down to 60 or 65 deg. by the addition of ice. When the temperature in the return falls to 100 deg., or should shivering occur, he must be taken out, dried, and put to bed. This treatment may be repeated when necessary. Stimulants are required both before and after immersion in the bath, and are best given in the form of brandy. If the hyperpyrexia is only slight, cold sponging may suffice to reduce the temperature.

Respiratory complications may be treated on general principles, combined with free stimulation.

Should pneumonia occur with recession of the joint inflammation, the application of a blister to the joint is followed as a rule by a marked amelioration of the respiratory complication.

It is of the utmost importance that the heart be examined daily, and should signs of peri or endo-cardial inflammation appear, a succession of blisters should be applied over the cardiac area. Stimulants, too, are generally called for. In the event of any cardiac complication occuring the patient must be confined to bed for several weeks at least, and the greatest care must be taken when he is allowed to get up. Prolonged rest, and the judicious employment of cardiac tonics do much to obviate the distressing symptoms which are so frequently observed to follow the occurrence of heart complications in acute rheumatism.

